# Platelet concentrate and type II IL-1 receptor are risk factors for allergic transfusion reactions in children

**DOI:** 10.1186/s13052-020-00869-6

**Published:** 2020-07-29

**Authors:** Wenjing Hu, Li Feng, Meng Li, Ting Li, Yudong Dai, Xiaowei Wang

**Affiliations:** 1 Department of Blood Transfusion, Women’s Hospital of Nanjing Medical University, Nanjing Matcrnity and Child Health Care Hospital, Nanjing, 210004 China; 2 Department of Blood Transfusion, Children’ s Hospital of Nanjing Medical University, 72 Guangzhou Avenue, Nanjing, Jiangsu 210008 P.R. China; 3 Nanjing Red Cross Blood Center, #3 Zizhulin, Nanjing, 210003 China

**Keywords:** Type II IL-1 decoy receptor, Allergic transfusion reactions, Platelet concentrate, Logistics regression analysis

## Abstract

**Purpose:**

Allergic transfusion reactions (ATRs) are immunological reactions after transfusion. Interleukin-1 (IL-1) is a critical regulator for human diseases. We performed this study to investigate the association of type II IL-1 decoy receptor (IL1R2) expression with ATRs in children.

**Methods:**

Children received blood transfusions between January and December 2019 were included. The age, sex, number and type of blood transfusion, allergic history, and medical history were collected and statistically analyzed. The blood samples were collected from children with and without ATRs for detecting the relative expression IL1R2 mRNA. Logistics regression analysis was performed to identify the risk factors for ATRs in children. The area under the receiver operating characteristic (ROC) curve (AUC) was used to evaluate the predictive performance of risk factors.

**Results:**

Totally, 28,840 transfusions in 20,230 children, with 236 ATRs (0.82%) in 117 patients (0.58%) were included. ATRs were common in children at the hematology-oncology department, in children received higher number of blood transfusions, and older children. Platelet concentrate induced a higher incidence of ATRs (3.31%) than red cell concentrate (0.22%, *p* < 0.0001). After the transfusion, IL1R2 mRNA level was higher in the blood samples in children with ATRs than those without ATRs (p < 0.0001). Logistics regression analysis indicated that platelet concentrate (95% CI 3.555, 293.782) and IL1R2 expression (95% CI 1.171 × 10^2^, 1.494 × 10^4^) were independent risk factors for ATRs in children. IL1R2 expression had high performance in predicting ATRs (AUC = 0.998, 100% sensitivity and 98.85% specificity).

**Conclusion:**

High IL1R2 expression level in children who received blood transfusions may predict the morbidity of ATR.

## Highlights

1. The frequency of allergic transfusion reactions (ATRs) post blood transfusion is 0.82%.
2. Platelet transfusion induced a higher ATRs incidence (3.31%) than the 0.22% of red cells.
3. Patients with ATRs had a higher level of IL1R2 mRNA than control.
4. IL1R2 expression was an independent risk factor for ATRs in children.

## Introduction

Allergic transfusion reactions (ATRs) are the most common complications of blood transfusions, especially for the platelet transfusion [1]. Platelet has been identified as a risk factor for ATRs [2]. However, the molecular mechanisms underlying ATRs are largely unknown at present.

ATRs are immunological reactions mediated by the allergen-dependent and -independent pathways [1]. The release of histamine and platelet-activating factor (PAF) are involved in allergen-dependent pathways [1]. Interleukin-1 (IL-1) stimulates PAF production in human vascular endothelial cells [3, 4], and the latter enhances the production of tumor necrosis factor-alpha (TNF-α) and IL-1 in monocytes [5]. Therefore, PAF may be the major chemical mediator and the biggest culprit of ATRs. What’s more, the association of cytokines including IL-6 and IL-8 with ATRs and febrile non-hemolytic transfusion reactions (FNHTR) in pediatric patients had been confirmed [6].

IL-1 polypeptides, including IL-1α and IL-1β, are critical activators and regulators for the pathogenesis of human diseases [7]. There are two types of IL-1 receptors, the type I IL-1 receptor (IL1R1) which is responsible for the transduction of IL-1-dependent intracellular signaling; and the type II IL-1 receptor (IL1R2) that lacks the IL-1 intracellular TIR signal transduction domain, on contrast, serves as a decoy receptor and an inhibitor of IL-1 signaling [7]. IL1R2 may act through hindering and neutralizing the IL-1 signal transduction [8, 9]. IL1R2 is natively expressed in neutrophils, monocytes, and macrophages, and is inducible in endothelial cells [10]. The expression of IL1R2 has been identified as a potential therapeutic target for several diseases including arteriosclerosis [7, 8]. However, the association of IL1R2 expression with ATRs in children is not clear till now.

We performed this study to investigate the difference in IL1R2 expression in pediatric patients with and without ATRs after transfusion. The potential of using IL1R2 as an independent risk factor for ATRs would be statistically analyzed and discussed. This study may provide more information on the pathogenesis and management of ATRs in children.

## Materials and methods

### Ethical statement

Ethical approval was obtained from the Ethics Committees of the Children’s Hospital of Nanjing Medical University, Nanjing, China. Human experiments were performed strictly according to the revised (2013) Helsinki Declaration of 1975. Written informed consents were obtained from the guardians of all children before the collection of the blood samples.

### Subjects and baseline characteristics

This study included children (aged ≤18) who received blood transfusion therapy during January and December 2019 at our hospital. Transfusion products included platelet concentrate (PC, γ irradiation-treated), suspended leukocyte-reduced red blood cells (SLRBC), fresh-frozen plasma (FFP), and cryoprecipitate. All the blood products were obtained from Nanjing Red Cross Blood Center, Nanjing, China. The baseline characteristics including age, sex, number, and type of transfusion received, history of allergy, and medicine were recorded. All children received premedication before blood transfusion.

### FNHTR and ATR definition

ATRs and related inflammatory symptoms were defined and reviewed by the physicians and/or nurses with reference to the clinical course and medical history of patients. FNHTR was defined as fever (body temperature rose more than 1 °C within 24 h) which may be companied by nausea, rigor, vomiting, skin itching, transient hypertension, and dyspnea without hemolysis and bacterial infection. Acute ATRs were assessed and defined as patients with at least one symptom within the first 4 h after transfusion, including skin itching, urticaria, rash, fever, rigor, jaundice, cough, dyspnea, bronchospasm, stomachache, headache, conjunctival edema, erythema, edema of the periorbital area, angioedema at the transfusion site, and hypotension [11]. Both FNHTRs and acute ATRs were categorized as ATRs in this study.

### Data classification

The data from pediatric patients were classified and grouped according to age (< 3 years, 3–6 years, 6–12 years and 12–18 years), sex, the number of transfusions received (1, 2–5 times, 6–10 times and > 11 times), product type (PC, SLRBC, FFP, and cryoprecipitate), allergic and medical history (food, pollen, rhinitis, and drug), department and ATR types.

### Blood collection and real-time RT-PCR analysis

Venous blood samples were collected into BD PAXgene Blood RNA tube (Becton, Dickinson and Company, NJ, USA) from patients before premedication and after blood transfusion (within 24 h post transfusion or in the first 24 h of ATRs). Blood samples were stored at − 20 °C before RNA extraction. Total RNA was isolated using QIAamp RNA Blood Mini Kits (Qiagen, Nanjing, China) following the protocol from manufacturers. RNA samples were reversely transcribed into the first-strand cDNA and double-strand DNA. The amplification of IL1R2 mRNA was performed using an SYBR Green Master Mix kit (Vazyme, Nanjing, China) and the specific primers (IL1R2 forward: 5′-ACCGCTGTGTCCTGACATTT− 3′ and reverse: 5′-GGAAGAGCGAAACCCACAGA-3′). RT-PCR analysis was conducted according to the reaction conditions: 95 °C for 4 min; 40 cycles of 95 °C for 20 s, 58 °C for 30 s and 72 °C for 20 s; and 72 °C for 5 min. The relative expression level of IL1R2 mRNA was calculated using the 2^−△△Ct^ method. GAPDH was used as the internal reference gene.

### Statistical analysis

The SPSS 22.0 software was used for the statistical analysis. The frequency of ATRs was presented as proportion and compared using Fisher’s exact test. Logistics regression analyses were performed to identify the risk factors for ATRs. Odds ratio (OR) and 95% confidence interval (CI) were calculated. The relative expression level of IL1R2 mRNA was expressed as the mean ± standard deviation. The one way ANOVA test (with Tukey test) was used to analyze the difference in the relative expression level of IL1R2 mRNA. The accuracy for predicting ATRs in children was evaluated using the area under the receiver operating characteristic (ROC) curve (AUC). *P* < 0.05 was set as the threshold for statistical significance.

## Results

### Demographic characteristics

A total of 28,840 transfusions and 20,230 children were included in this study, with an average number of 1.43 transfusions per child. Two-hundred and thirty-six ATRs (0.82%, 236/28840) in 117 patients (0.58%, 117/20,230) were reported. There was no difference in the frequency of ATRs between female and male children (*p* = 0.514), and children with and without a history of allergy (*p* = 0.134; Table 1). Children at Hematology-Oncology ward ranked the first in the frequency of ATRs (1.59%), followed by children in surgical ICU department (0.88%). A higher number of blood transfusions and older age significantly increased the risk of ATRs (*p* < 0.0001). In addition, children received PC were at a higher risk of ATRs (3.31%) compared with patients received SLRBC (0.22%), FFP (0.45%), and cryoprecipitate (2.27%; *p* < 0.0001). Urticaria ranked first in the type of ATR (68.64%), followed by rash (6.78%), and erythema (6.78%). Fourteen children had FNHTRs (5.93%), and 20 children (8.47%) had the other types of ATRs (including 4 coughs, 2 dyspnea, 1 rigor, 2 stomachaches, 3 laryngeal edema, 2 shocks, 2 emeses, and 4 asymptomatic itches).

**Table 1:** The demographic characteristics of all children

Variable	Number of patients/transfusion	Number of ATRs	P
Sex	*n* = 20,230	*n* = 117	0.514
-Female	9161	49 (0.53%)	
-Male	11,069	68 (0.62%)	
Age	n = 20,230	n = 117	< 0.0001
- < 3 years	9332	18 (0.19%)	
-3-6 years	8350	55 (0.66%)	
-6 − 12 years	1364	29 (2.13%)	
−12-18 years	1184	15 (1.27%)	
Allergic history	n = 20,230	n = 117	0.134
-None	18,395	102 (0.55%)	
-Drug	651	7 (1.08%)	
-Food, Rhinitis, pollen and others	1184	8 (0.68%)	
Number of transfusion per patient	n = 20,230	n = 117	< 0.0001
-1	7816	21 (0.27%)	
−2-5	9751	56 (0.57%)	
−6-10	2195	61 (2.78%)	
- > 11	468	13 (2.78%)	
Products	*n* = 28,840	*n* = 236	< 0.0001
-PC	4292	142 (3.31%)	
-SLRBC	11,571	26 (0.22%)	
-FFP	12,801	64 (0.45%)	
-Cryoprecipitate	176	4 (2.27%)	
Department	n = 28,840	n = 236	< 0.0001
-Hematology-Oncology	6814	109 (1.59%)	
-Surgical ICU	2162	19 (0.88%)	
-Pediatric ICU	1876	5 (0.27%)	
-Cardiothoracic surgery	3139	18 (0.57%)	
-General surgery	2697	21 (0.78%)	
-Outpatient	9321	47 (0.50%)	
-Other	2831	17 (0.60%)	
ATR type		n = 236	
-urticaria	/	162 (68.64%)	
-FNHTR	/	14 (5.93%)	
-rash	/	16 (6.78%)	
-erythema	/	16 (6.78%)	
-skin itching	/	8 (3.39%)	
-Others	/	20 (8.47%)	

*ATR* allergic transfusion reaction, *PC* platelet concentrate, *SLRBC* suspended leukocyte-reduced red blood cells, *FFP* fresh-frozen plasma, *ICU* intensive care unit, *FNHTR* non-hemolytic transfusion reactions

### The different expression level of IL1R2 in patients with and without ATR

**Fig. 1:**
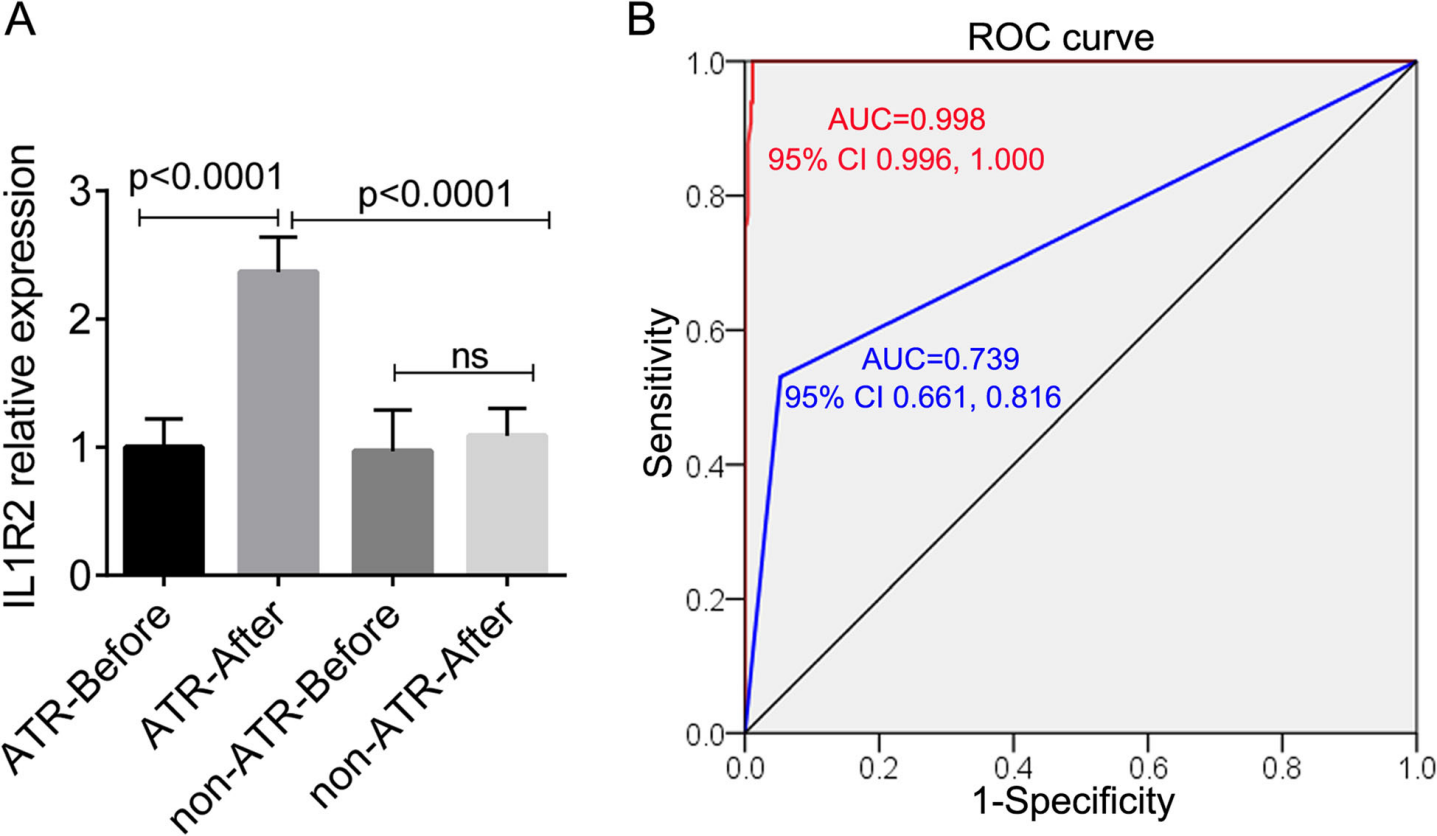
The relative expression level of IL1R2 mRNA in children with and without allergic transfusion reactions (ATRs; **a**) and the receiver operating characteristic (ROC) curve analysis (**b**). The blood samples were collected before premedication and after blood transfusion (within 24 h post transfusion or in the first 24 h of ATRs). **a**, the relative expression level of IL1R2 mRNA in the blood samples in children with and without ATRs was detected using RT-PCR analysis. Ns, not significant. **b**, the ROC analysis for predicting ATRs using platelet concentrate (blue line; AUC = 0.739) and IL1R2 expression (red line; AUC = 0.998). AUC, the area under the ROC curve

The blood samples were collected from children before premedication and after blood transfusion, including 66 patients with ATRs (including 6 FNHTRs). The paired blood samples were taken from 66 with ATRs and 500 patients without ATRs before premedication and after blood transfusion, respectively, were used for PCR analysis. In children with ATRs, the relative expression level of IL1R2 was significantly upregulated after blood transfusion versus before (*p* < 0.0001; Fig. 1A), and was higher than children without ATRs (*p* < 0.0001). No significant changes were observed in the expression level of IL1R2 mRNA in children without ATRs before and after transfusion.

### Identification of risk factors for ATR

Univariate logistics regression analysis showed that PC (OR = 20.175, 95% CI 10.635, 38.275, *p* < 0.0001; Table 2), number of transfusion (OR = 1.648, 95% CI 1.426, 1.904, *p* < 0.0001), age (OR = 1.076, 95% CI 1.016, 1.139, *p* = 0.013), and IL1R2 expression (OR = 5.574 × 10^3^, 95% CI 2.569 × 10^2^, 5.756 × 10^4^, *p* < 0.0001) were associated with ATRs in children. Multivariate logistics regression analysis indicated that PC (OR = 32.318, 95% CI 3.555, 293.782, *p* = 0.002) and IL1R2 expression (OR = 1.322 × 10^3^, 95% CI 1.171 × 10^2^, 1.494 × 10^4^, *p* < 0.0001) were independent risk factors for ATRs (Table 2). After removing the FNHTRs cases (*n* = 6), PC and IL1R2 expression were still the risk factors for ATRs (*p* < 0.01, Table S1). The ROC analysis showed that IL1R2 expression (AUC = 0.998, 95% CI 0.996, 1.000, p < 0.0001) and PC (AUC = 0.739, 95% CI 0.661, 0.816, p < 0.0001) had high performance for predicting ATRs in children (Fig. 1B). The sensitivity and specificity of IL1R2 expression and PC was 100% (95% CI 93.15, 100%) and 60.34% (95% CI 46.64, 72.68%), and 98.85% (95% CI 97.17, 99.58%) and 92.99% (95% CI 90.09, 95.11%), respectively.

**Table 2:** Logistics regression analysis of the risk factors for allergic transfusion reactions

Variable	β	Univariate OR (95% CI)	P	β	Multivariate OR (95% CI)	P
Sex	−0.342	0.710 (0.421,1.198)	0.200			
PC	3.004	20.175 (10.635, 38.275)	< 0.0001	3.476	32.318 (3.555, 293.782)	0.002
Number of transfusion	0.499	1.648 (1.426, 1.904)	< 0.0001	0.152	1.164 (0.646, 2.099)	0.613
Age	0.073	1.076 (1.016, 1.139)	0.013	0.085	1.089 (0.877, 1.352)	0.440
IL1R2 expression	8.255	5.574 × 10^3^ (2.569 × 10^2^, 5.756 × 10^4^)	< 0.0001	7.187	1.322 × 10^3^ (1.171 × 10^2^, 1.494 × 10^4^)	< 0.0001

*OR* odds ratio, *CI* confidence interval, *PC* platelet concentrate

## Discussion

The association of PC transfusion and cytokines of IL − 6 and IL-8 with ATRs has been reported in children [6]. PAF-mediated allergen-dependent pathway during ATRs may play a major role and act as the biggest culprit of the pathogenesis of ATRs [4]. The modulation of IL-1 on PAF or vice versa may provide evidence on IL-1-mediated inflammation in ATRs [3–5]. Our present study demonstrated that the expression of IL1R2 mRNA in children with ATRs was elevated post transfusion. We showed that PC transfusion and IL1R2 expression were independent risk factors for ATRs.

PC transfusion has been identified as a leading cause of transfusion reactions [12]. Here in our study, the frequency of ATRs post PC transfusion was 3.31%, and that post SLRBC transfusion was 0.22%. This data was consistent with the reported ATRs frequency of less than 6% in previous reports [13, 14]. As expected, PC transfusion was identified as a risk factor for ATRs after transfusion (95% CI 3.555, 293.782). We confirmed that PC transfusion had a high accuracy in predicting ATRs in children with a relatively high specificity and sensitivity. The reduced utilization of PC would decrease the incidence of ATRs.

Poststorage leukoreduction of blood products, including apheresis platelets, significantly increased the production of pro-inflammatory cytokines like IL-1β and IL-8. This fact was associated with the increased incidence of ATRs to the transfusion of poststorage leukocyte-reduced blood components [15]. Lin et al. also reported the significant increase in the levels of plasma IL-8 and IL-6 in patients had FNHTR [16]. Our present study for the first time reported the significant increase of IL1R2 mRNA in children with ATRs post transfusion. The increased expression level of IL1R2 was an independent risk factor for ATRs in children.

It was previously reported that FNHTRs were associated with the increased levels of human leukocyte antigen (HLA) antibodies, and ATRs were related to the deficiency of antibodies including IgA, IgG, and IgE [17]. However, the associations are not common now because of the use of washed and filtered RBC, and irradiation-treated platelets [17, 18]. A previous report has shown that HLA-DR mediated the production of IL-1β in human monocytes [19]. Also, there is a contrary regulation that IL-1β upregulated HLA-G expression in immune cells [20]. The activation of IL-1β-dependent intracellular signaling plays crucial role in immune diseases and responses [7, 21, 22]. However, there is less information on the association of IL1R2 with IL-1β-dependent intracellular signaling during the pathogenesis of diseases. The increased expression of IL1R2 has been associated with poor prognosis and metastasis of several malignancies [23, 24]. The ratio of IL-1β/IL1R2 positively correlated with the production of interferon γ, and negatively correlated with TNF-α production in women with invasive ductal mammary adenocarcinomas [23]. These results showed that the dynamic equilibrium between IL-1-dependent intracellular signaling and IL1R2 was associated with the progression of human diseases.

IL-1-dependent intracellular signaling attributes to the pathogenesis of many diseases including arteriosclerosis [8], rheumatoid arthritis [25], type 2 diabetes [25, 26], and pustular psoriasis [27]. The inhibition of IL-1, however, showed an impressive effect on treating these diseases [28, 29]. Upon the activation of IL, IL1R2 competes with IL1R1 for IL-1 and forms an IL1R2/IL-1RAP complex, and therefore suppressing IL-1-dependent intracellular signaling [7]. In in vitro and in vivo experiments, expression of IL1R2 has been identified as an anti-inflammatory mediator with therapeutic value in several diseases including arteriosclerosis [7, 8] and arthritis [30, 31]. According to the above studies, we speculated that the significant upregulation of IL1R2 in children with ATRs might act on suppressing IL-1 signaling and reducing the inflammatory responses in patients.

## Conclusions

We concluded that the expression of IL1R2 and platelet transfusion could be used as independent factors for ATRs in children that received blood transfusions. The high expression of IL1R2 in children with ATRs might be an indicator of suppressing IL-1-mediated inflammations.

## Supplementary information

**Additional file 1: Table S1.** Logistics regression analysis of the risk factors for allergic transfusion reactions without febrile non-hemolytic transfusion reactions.

## Data Availability

The data supporting the conclusions of this article are included within the article. The original data is available from author (Xiaowei Wang) with reasonable request.
