# Supplementary material for: Platelet concentrate and type II IL-1 receptor are risk factors for allergic transfusion reactions in children
Source: Ital J Pediatr. 2020 Jul 29;46:109. doi: 10.1186/s13052-020-00869-6 (PMC7392823; doi:10.1186/s13052-020-00869-6)
Supplement: Supplementary file 1 — Additional file 1: Table S1. Logistics regression analysis of the risk factors for allergic transfusion reactions without febrile non-hemolytic transfusion reactions. [file 13052_2020_869_MOESM1_ESM.docx]

Table S1. Logistics regression analysis of the risk factors for allergic transfusion reactions without febrile non-hemolytic transfusion reactions.

| Variable |  | Univariate |  |  | Multivariate |  |
| --- | --- | --- | --- | --- | --- | --- |
|  | β | OR (95% CI) | P | β | OR (95% CI) | P |
| Sex | -0.343 | 0.709 (0.421,1.199) | 0.200 |  |  |  |
| PC | 3.004 | 20.172 (9.376, 36.944) | <0.0001 | 3.198 | 29.674 (3.005, 300.584) | 0.002 |
| Number of transfusion | 0.499 | 1.645 (1.416, 1.903) | <0.0001 | 0.155 | 1.164 (0.667, 2.019) | 0.608 |
| Age | 0.068 | 1.178(1.016, 1.296) | 0.037 | 0.089 | 1.088(0.877, 1.334) | 0.432 |
| IL1R2 expression | 8.261 | 5.585×10^3^ (2.571×10^2^, 4.655×10^4^) | <0.0001 | 7.699 | 1.202×10^3^ (1.101×10^2^, 1.271×10^4^) | <0.0001 |

OR, odds ratio. CI, confidence interval. PC, platelet concentrate. FNHTR, febrile non-hemolytic transfusion reactions.
